# Adherence to malaria diagnosis and treatment guidelines among healthcare workers in Ogun State, Nigeria

**DOI:** 10.1186/s12889-016-3495-x

**Published:** 2016-08-19

**Authors:** Oluyomi F. Bamiselu, IkeOluwapo Ajayi, Olufunmilayo Fawole, David Dairo, Olufemi Ajumobi, Abisola Oladimeji, Yoon Steven

**Affiliations:** 1 Nigeria Field Epidemiology and Laboratory Training Programme, Abuja, Nigeria; 2 Department of Epidemiology and Medical Statistics, Faculty of Public Health, University of Ibadan, Ibadan, Nigeria; 3 National Malaria Elimination Programme, Abuja, Nigeria; 4 Division of Parasitic Diseases and Malaria, Center for Global Health, Centers for Disease Control and Prevention, Atlanta, Georgia

**Keywords:** Malaria, National treatment guidelines, Healthcare workers’ adherence, Nigeria

## Abstract

**Background:**

Malaria case management remains a vital component of malaria control strategies. Despite the introduction of national malaria treatment guidelines and scale-up of malaria control interventions in Nigeria, anecdotal evidence shows some deviations from the guidelines in malaria case management. This study assessed factors influencing adherence to malaria diagnosis and treatment guidelines among healthcare workers in public and private sectors in Ogun State, Nigeria.

**Methods:**

A comparative cross-sectional study was carried out among 432 (216 public and 216 private) healthcare workers selected from nine Local Government Areas using a multistage sampling technique. A pre-tested interviewer administered questionnaire was used to collect information on availability and use of malaria Rapid Diagnostic Test (mRDT) and artemisinin combination therapy (ACT), for management of uncomplicated malaria. Adherence was defined as when choice of antimalarials for parasitological confirmed malaria cases was restricted to recommended antimalarial medicines. Association between adherence and independent variables were tested using Chi-square at 5 % level of significance.

**Results:**

Malaria RDT was available in 81.9 % of the public health facilities and 19.4 % of the private health facilities (*p* = 0.001). Its use was higher among public healthcare workers (85.2 %) compared to 32.9 % in private facilities (*p* = 0.000). Presumptive diagnosis of malaria was higher among private healthcare workers (94.9 %) compared to 22.7 % public facilities (*p* = <0.0001). The main reason for non-usage of mRDT among private healthcare workers was its perceived unreliability of mRDT (40.9 %). Monotherapy including artesunate (58.3 % vs 12.5 %), amodiaquine (38.9 % vs 8.3 %) and chloroquine (26.4 % vs 4.2 %) were significantly more available in private than public health facilities, respectively. Adherence to guidelines was significantly higher among public healthcare workers (60.6 %) compared to those in private facilities (27.3 %). Availability of antimalarial medicine was the main factor that influenced treatment prescription in both healthcare settings (*p* = 0.27). However, drug promotion by manufactures (45.8 %) has a major influence on private healthcare workers’ prescription practice.

**Conclusion:**

The findings of this study demonstrate significant difference between public and private healthcare workers on adherence to national malaria diagnosis and treatment guidelines. Interventions to improve private sector engagement in implementation of the guidelines, training and supply of recommended antimalarial medicines should be intensified.

## Background

Malaria is a complex disease that differs in epidemiology and public health impact in different parts of the world. It affects 3.4 billion people, about half of the world’s population in 104 countries and territories [1]. The World Health Organisation (WHO) estimates that 198 million cases of malaria occurred globally in 2013 with 584 000 deaths [1]. In sub-Saharan Africa, 18 countries account for 80 % of global.malaria infections [1]. Most cases (82 %) and deaths (90 %) occur in Africa, and most deaths (78 %) are in children under 5 years of age [1]. About 90 % of global malaria deaths occur in 30 countries in sub-Saharan Africa [1]. Nigeria and the Democratic Republic of the Congo together accounted for 39 % of the global total of estimated malaria deaths and 34 % of cases in 2013 [1].

Malaria is a major public health problem in Nigeria, accounting for about 60 % of all outpatient attendances and 30 % of all hospital admissions [2]. It is estimated that malaria is responsible for nearly 110 million clinical cases and an estimated 300,000 deaths per year, including up to 11 % of maternal mortality [2]. Malaria’s economic impact is enormous with about N132 billion lost to malaria annually in form of treatment costs, prevention and loss of man hours among other control costs [2]. In Ogun State, malaria is responsible for about 70 % of outpatient attendance at the secondary healthcare facilities and over 80 % of all cases seen in primary healthcare facilities [3]. In 2012, there were 116,372 reported malaria cases in the State with an annual incidence rate of 26/1000 population [3].

Prompt and effective case management of uncomplicated malaria is a critical element of malaria control. These involve accurate clinical assessment, laboratory confirmation of malaria either by malaria rapid diagnostic test (mRDT) or microscopy prior to treatment with an effective antimalarial [4]. Introduction of Artemisinin-based combination therapy (ACT) and rapid diagnostic test (RDT) have improved malaria case management substantially. However, development and spread of artemisinin resistance may have dire consequences for the recent achievements in malaria control if health care providers do not adhere to standard diagnostic and treatment guidelines. Thus, it is important to change from symptom-based presumptive treatment to parasitological confirmation of malaria infection before initiation of antimalarial treatment. The use of parasite-based diagnosis allows for efficient utilisation of anti-malarial medicines, and provides an opportunity for other causes of fever to be identified early and treated appropriately [5].

Although the awareness level of the new treatment policy has improved over time, studies have shown that other factors militate against adherence to the national malaria treatment guidelines by healthcare providers [6–8]. Some studies have shown marked variations between health care providers, with clinicians consistently demonstrating retention and use of knowledge and good practice [9–11]. The findings from a study conducted in Mozambique in 2011 demonstrated that public health care workers’s adherence to the national guideline for malaria treatment was still poor and prescription of ACT to malaria negative patients remained high [12]. The implementation of effective case management may result in a number of challenges, of which availability of commodities at health facilities and sub-optimal case-management practices are of particular concern. In Nigeria, a study that assessed availability and use of mRDTs in public and private health facilities found limited use of mRDTs in these facilities [11]. The factors that influence case management in both the private and public sector may differ. Some studies in Nigeria reported that primary health care providers had fairly adequate knowledge on basic concepts of malaria but treatment practices were poor [13]. A study conducted in South Eastern Nigeria in 2014 revealed that utilization of ACTs for treating uncomplicated malaria in the States has improved but laboratory confirmation of diagnosis were suboptimum [14]. There is paucity of data on the management of malaria cases provided at health facilities in Ogun state. Clinicians in the private sector are often assumed to use more irrational treatments compare to those in the public sector [15]. The knowledge of health workers’ perceptions of the usefulness of RDTs and the influence on their drug prescription practices will provide useful information for promoting effective and improved malaria management. This study was therefore conducted to determine adherence to malaria diagnosis and treatment guideline among health care service providers in public and private sectors in Ogun State, southwest, Nigeria.

## Methods

### Study area

The study was conducted in Ogun State, southwest Nigeria. Ogun State comprises of 4,624,756 individual disaggregated into 2,294,523 males and 2,330,233 females [16]. The greater proportion of the State lies in the tropical rainforest zone, while the northern part has features of Guinea savannah. The State experiences malaria transmission all year-round with peak transmission during rainy season. The State has three senatorial zones, 20 Local Government Areas (LGAs) and 236 political wards and operates a three-tier health care delivery services namely primary, secondary and tertiary spread across urban and rural areas. The primary healthcare is the first level of contact with individuals and family while the secondary healthcare refers to a second tier of health system, in which patients from primary healthcare are referred to specialist in higher hospitals for treatment. The tertiary healthcare refers to a third level of health system, in which specialized consultative care is provided usually on referral from primary and secondary medical care. The State has a mix of both public and private health facilities totaling 1280 health facilities; disaggregated into 450 Primary health centers (PHCs), 39 secondary facilities, three tertiary facilities (1 State and 2 Federal) and 790 registered privates facilities. Apart from few health facilities that offer only specialized care such as Dental hospitals and Eye clinics, majority of the facilities in the State offer malaria diagnosis and treatment.. Generally, trained staff of public primary health centres/private sector equivalent offer malaria diagnosis using RDT while trained laboratory scientists at public general hospitals/private sector equivalent (secondary care level) offer malaria microscopy and RDT services. Moreover, there are trained staff of these general hospitals/private sector equivalent that can perform RDT. Public health workers were persons employed by the government in tertiary hospitals, secondary hospitals and primary health care workers while private health workers were those employed by health facilities owned by individuals, religious missions and non-governmental organizations (NGOs). It is estimated that about 60 % of people in the State seek medical care in the private health facilities [3].

### Study design

Comparative cross sectional study involving health care providers in public and private health care facilities was carried out. The study was conducted between March and April, 2015.

### Sample size and sampling technique

**Fig. 1:**
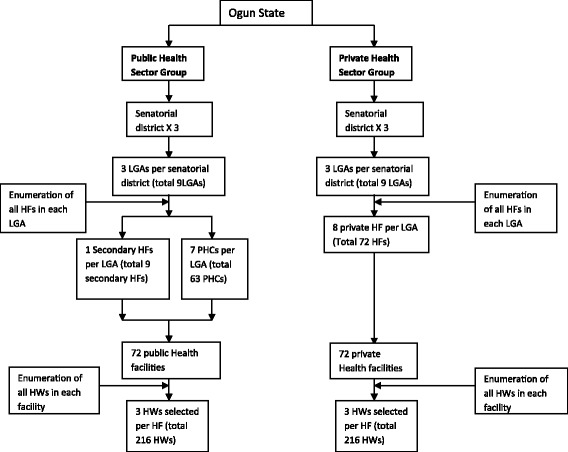
Flow chart of recruitment of respondents

A total of 432 health workers comprising 216 each in the public and private health facilities participated in this study. They were selected using multistage sampling technique. From each of the three senatorial districts in the State, three LGAs were chosen by random sampling procedure to make a total of nine LGAs. In each of the selected LGA, the health facilities were stratified based on type into public and private, and eight public and private health facilities were selected by ballot from each LGA to make a total of 16 health facilities per LGA. The public health facilities were furthered stratified into primary and secondary health facilities. Overall, 144 health facilities, comprising of 72 each in private and public health sectors were selected. At each health facility, three healthcare workers were selected randomly from the list of all eligible healthcare workers obtained. An eligible worker was one who offered malaria treatment either in public or private health facilities. Overall, 432 healthcare workers were interviewed. Figure 1 shows the flow chart of the sampling method.

### Data collection

Volunteer personnel from Epidemiology unit of Ministry of Health and Local Government Area (LGA) Disease Surveillance and Notification Officers (DSNOs) in the nine selected LGAs were recruited and trained as interviewers. They administered pre-tested semi-structured questionnaires to study participants to obtain information on respondents socio-demographics characteristics, level of awareness of national malaria treatment guideline, availability of mRDTs and ACTs for diagnosis and treatment of malaria, level of adherence to mRDT and ACT use in the health facilities and factors influencing adherence to malaria treatment guidelines. On adherence to use of mRDTs and ACTs the questions were based on current utilization of mRDTs and prescription of antimalarial medicine for diagnosis and treatment of uncomplicated malaria cases respectively. A health facility observational checklist was also administered by interviewer to assess availability of mRDTs, microscopes and the types of antimalarial medicines available at the facilities within the last 3 months.

### Data processing and analysis

The dependent variable was adherence of healthcare worker in public and private health sectors to national treatment guideline for management of uncomplicated malaria. The degree of adherence was classified as strict, partial and nonadherence. Strict adherence was whenther choice of antimalarial medicines for treatment of parasitological confirmed malaria cases were restricted to those recommended in the national guideline for malaria treatment; partial adherence was when there was no parasitological confirmation of cases but choice of antimalarials conformed to recommended national guideline for malaria treatment and nonadherence, when there was no parasitological confirmation of cases and the choice of antimalarial medicines did not follow the recommendations of the national malaria treatment guideline. Independent variables were categorized into health workers and health facility-related factors influencing adherence to national treatment and diagnostic guidelines. Health workers’ factors identified were socio-demographic characteristic, cadre of health workers, health workers’ knowledge about malaria case management, and access to national guidelines. Health facility factors include availability of functional diagnostic equipment (mRDT and microscope), antimalarial medicines and the national guidelines. Availability of diagnostic equipment and anti-malarial medicines was measured as the proportion of health facilities with at least one functional malaria diagnostic equipment and recommended anti-malarial medicine in stock in the last 3 months before the survey.

Data entry, cleaning and analysis were done using Epi info 3.5.2. The data were cleaned and checked for completeness and outliers before analysis. Descriptive statistics (frequencies, proportions, percentages, means and standard deviations), tables and charts were used to summarize the data. Association between the adherence and independent variables were tested using Chi-square and Fisher’s tests and results were considered significant at *p* < 0.05.

### Ethical considerations

This research was approved by the Ethics and Research Committee of Ogun State Ministry of Health (Reference number - PH/200/31; Date – 28/11/2014). Participation was voluntary and written informed consent was obtained from all respondents. The participants were not at any point in time exposed to harm as a result of their participation and were free to withdraw their participation at any time during the interview. Confidentiality of collected information was maintained by using non personal identifier codes for the respondents. The completed questionnaire was kept under lock and key.

## Results

### Characteristics of respondents

The socio-demographic characteristics of the respondents are summarized in Table 1. A total of 432 respondents participated in the survey, representing 216 health workers each from public and private health facilities. Overall, majority of the health workers interviewed were females (69.9 %). However, in the private health facilities male respondents (56.9 %) were more than females (Table 1). The mean age of the respondents was 40.6 years (SD: 8.59). The average age of public and private health workers was 42.1 (SD: 8.4) and 39.1 (SD: 8.6) years, respectively. Majority of respondents from public health facilities (42.3 %) were aged 45-54 years, while most (44.2 %) from private health facilities were aged 35–44 years. Seven eight percent of health workers in public health facilities mentioned they have been trained on malaria case management while only 24 % of providers in private setting had been trained (Table 1).

**Table 1:** Characteristics of the healthcare workers, Ogun State, Nigeria

	Public facilities *N* = 216 n (%)	Private facilities *N* = 216 n (%)	Total *N* = 432 n (%)
Gender			
Male	37 (17.1)	93 (43.1)	130 (30.1)
Female	179 (82.8)	123 (56.9)	302 (69.9)
Age (years)			
< 25	4 (1.9)	6 (2.8)	10 (2.3)
25–34	36 (16.7)	64 (29.6)	100 (23.1)
35–44	79 (36.6)	95 (43.9)	174 (40.3)
45–54	89 (41.2)	40 (18.5)	129 (29.9)
≥ 55	8 (3.7)	11 (5.1)	19 (4.4)
Cadre of Health workers			
Consultant	0	4 (1.9)	4 (0.9)
Medical Officer	25 (11.6)	110 (51.6)	135 (31.5)
Nurse/Midwife	99 (46.0)	92 (43.2)	191 (44.6)
CHEW/CHO^a^	89 (41.4)	7 (3.3)	96 (22.4)
Laboratory Scientist	2 (0.9)	0	2 (0.5)
Years of practice			
1–5	21 (9.7)	43 (19.9)	64 (14.8)
6–10	37 (17.1)	60 (17.1)	97 (22.5)
11–15	32 ((14.8)	62 (28.7)	94 (21.8)
16–20	43 (19.9)	20 (9.3)	63 (14.6)
21–25	46 ((21.3)	14 (6.5)	60 (13.9)
> 25	37 (17.2)	17 (7.9)	54 (12.5)
Training on malaria case management			
Yes	170 (79.4)	52 (20.2)	222 (51.2)
No	46 (21.3)	164 (75.9)	210 (48.6)

*CHEW* Community Health Extension Worker; *CHO* Community Health Officers

^a^These cadre of health workere were group together because they are expected to work mainly at community level

### Characteristics of health facilities

Overall 50.7 % of the health facilities [81.9 % of public health facilities compared with 19.1 % of private health facilities] had mRDT kits in stock in the last 3 months prior to survey (Table 2). Seventeen percent and 25.0 % of the public and private facilities respectively used microscopy, (*p* = 0.22).

**Table 2:** Characteristics of the selected health facilities

	Public facilities *N* = 72 n (%)	Private facilities *N* = 72 n (%)	Total *N* = 144 n (%)	^a^OR (95 % CI)	*P*-value
Availability of malaria diagnostic equipment					
HF with microscopy	12 (16.7 %)	18 (25.0 %)	30 (20.8 %)	0.6 (0.3–1.4)	0.220
HF with mRDT	59 (81.9 %))	14 (19.4 %)	73 (50.7 %)	18.8 (8.1–3.4)	0.000
HF with both microscopy and mRDT	7 (9.7 %)	8 (11.1 %)	15 (10.4 %)	0.9 (0.3–2.5)	0.790
Availability of recommended antimalarial medicines					
AL	68 (94.4)	57 (79.2)	125 (86.8)	4.5 (1.4–14.2)	0.007
AA	58 (80.6)	52 (72.2)	110 (76.4)	1.6 (0.7–3.5)	0.240
Quinine tab	61 (84.7)	26 (36.1)	87 (60.4)	9.8 (4.4–21.9)	0.000
Quinine Inj.	43 (59.7)	18 (25.0)	61 (42.4)	4.5 (2.2–9.1)	0.000
Artemether Inj.	8 (11.1)	40 (55.6)	48 (33.3)	0.1 (0.0–0.2)	0.000
S-P	65 (90.3)	55 (76.4)	120 (83.3)	2.9 (1.1–7.4)	0.030
Non recommended antimalarial medicines					
Artesunate	9 (12.5)	42 (58.3)	51 (35.4)	0.1 (0.0–0.2)	0.000
Amodiaquine	6 (8.3)	28 (38.9)	34 (23.6)	0.1 (0.0–0.4)	0.0002
Chloroquine	3 (4.2)	19 (26.4)	22 (15.3)	0.1 (0.0–0.4)	0.002
Halofantrine	2 (2.8)	8 (11.1)	10 (6.9)	0.2 (0.1–1.1)	0.050
Dihydroartemisinin-piperaquine	2 (2.8)	5 (6.9)	7 (4.9)	0.4 (0.1–2.0)	0.250

^a^ *OR* Odd Ratio

Overall, artemether-lumefantrine (AL) was the most available (86.8 %) among all the recommended antimalarial medicines in the national malaria treatment guideline (Table 2). Artesunate-amodiaquine (AA) was available in 76.4 % of the facilities. Artemether-lumefantrine and AA were more often available in public facilities (94.4 % and 79.2 %) than in private facilities (79.2 % and 72.2 %), respectively. However, only the difference in availability of AL in the two facilities was significant. (*p* = 0.007). There were significant differences in the proportion of quinine tablet (84.7 % vs 36.1 %) and quinine injection (59.7 % vs 25.0 %) available in public and private facilities, respectively. A higher proportion of the public health facilities had sulfadoxine-pyrimethamine in stock (90.3 %) compared to the private facilities (76.4 %), (*p* =0.03).

Artesunate (monotherapy) was the most common of the non-recommended antimalarial medicines stocked in the facilities (35.4 %) with a significant difference in the proportion of private facilities (58.3 %) compared with public facilities (12.5 %). Twenty seven percent of the private health facilities stocked chloroquine in their facility, compared to 4.2 % public health facilities (*p* = 0.0002). Amodiaquine tablets were available in 38.9 % private health facilities and 8.3 % of public health facilities.

### Awareness of national malaria treatment guidelines and use of the guidelines in management of uncomplicated malaria

**Fig. 2:**
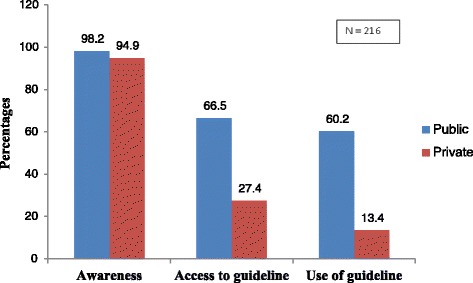
Awareness, accessibility and use of National malaria treatment guidelines among health workers in public and private health facilities in Ogun State, Nigeria

Of the 216 health care workers each from the public and private facilities, 212 (98.2 %) and 205 (94.9 %) were aware of the national malaria treatment guidelines, 144 (66.6 %) and 59 (27.3 %) had access to the guideline out of which 130 (90.3 %) and 29 (49.2 %) reported using the guidelines, respectively, *p* = 0.001 (Fig. 2).

### Providers’ knowledge of malaria case management

A high proportion (85.7 %) of the health workers interviewed in public health facilities stated the recommended doses of ACT for adults correctly compared to 66.7 % of health workers interviewed in private health facilities (*p* = 0.000). Almost all (95.7 %) health workers interviewed in public facilities and 93.5 % of health workers interviewed in private facilities responded correctly that food should be taken before using ACT (*p* = 0.40). However, 65.7 % and 47.3 % of the health workers in public and private health facilities, respectively knew that a fatty meal and milk are important for improving drug absorption of artemether lumenfantrine brand of ACT (*p* = 0.0002).

### Malaria diagnosis methods

Presumptive diagnosis was significantly more likely to be carried out in private health facilities (94.9 %) than public health facilities (22.7 %), (*p* = 0.000) while parasitological diagnosis using RDT was more likely to be used by health workers in public facilities (85.2 %) compared to private facilities (32.9 %), (*p* = 0.000). The main reasons reported by respondents for non-use of RDT were non-availability of RDT (42.3 %) among the public health workers and lack of confidence in RDT results (40.9 %) among health workers in private facilities.

### Adherence to national antimalarial treatment guideline when choosing antimalarials for treatment of parasitological confirmed malaria cases

Almost half of all the health workers interviewed (*n* = 186, 44.1 %) adhered strictly to the national treatment guideline in the choice of antimalarial medicines for parasitological confirmed malaria cases (Table 3). These included 129 (60.6 %) in public and 57 (27.3 %) in private health facilities. Sixty one (28.6 %) public health workers and 77 (36.8 %) private health workers partially adhered to the guideline when choosing antimalarials without parasitological confirmation. Non-adherence to prescription of recommended antimalarial medicines and laboratory confirmation was reported among 33.9 % of private health workers compared to 11.3 % of public health worker (*p* < 0.001).

**Table 3:** Adherence to the national malaria treatment guidelines by health workers when choosing medicines for malaria treatment

Level of adherence to malaria treatment guidelines	Public *N* = 206 n (%)	Private *N* = 205 n (%)	Total *N* = 411 n (%)	^a^OR (95 % CI)	*P*-value
Strictly adhered	129 (60.6)	57 (27.3)	186 (44.1)	4.10 (2.7–6.1)	0.000
Partially adhered	61 (28.6)	77 (36.8)	138 (32.7)	0.69 (0.5–1.2)	0.07
Did not adhere	24 (11.3)	71 (33.9)	95 (22.5)	0.25 (0.2–0.4)	0.000

^a^ *OR* Odd Ratio

### Health workers’ factors associated with adherence to national malaria treatment guideline

Table 4 – shows health workers’ factors associated with adherence to national treatment guideline. Health workers in both public (OR = 4.3, 95%CI = 1.7–10.9) and private (OR = 7.0, 95%CI = 3.3–14.7) health settings using malaria diagnostic method had significantly higher odds of adhering to the guideline. Training on malaria case management, access to the guidelines and availability of malaria diagnostic tool were not associated with adherence to the guidelines in both public and private health facilities.

**Table 4:** Health worker factors associated with adherence to national malaria treatment guidelines

Characteristics		Public health facilities			Private health facilities		
		^a^Adhere n (%)	Did not adhere n (%)	^b^OR (95 % CI)	Adhere n (%)	Did not adhere n (%)	OR (95 % CI)
Provider knowledge of medicine of choice for malaria treatment and dosing regimens	Had knowledge	127 (100)	52 (81.3)	-	71 (100)	69 (52.7)	-
	Didn’t have	0	23 (18.7)	Ref	0	62 (47.3)	Ref
Health workers who had access to the guidelines	Had access to guidelines	71 (64.6)	66 (66.7)	0.9 (0.5–1.6)	19 (26.0)	40 (34.2)	0.67 (0.4–1.3)
	Didn’t have	39 (35.5)	33 (33.3)	Ref	54 (73.9)	77 (65.8)	Ref
Health workers used any malaria diagnostic tool (RDT or microscopy)	Used diagnostic tool	128 (94.8)	68 (81.0)	4.3 (1.7–10.9)	65 (85.5)	49 (45.8)	7.0 (3.3–14.7)
	Didn’t use	7 (5.2)	16 (19.1)	Ref	11 (14.5)	58 (58.5)	Ref
Training on malaria case management	Received training	82 (73.9)	85 (84.2)	0.5 (0.3–1.1)	13 (18.1)	37 (27.2)	0.6 (0.3–1.2
	Didn’t receive	29 (26.1)	16 (15.8)	Ref	59 (81.9)	99 (72.8)	Ref

^a^Adhere’ is strict adherence: both parasitological diagnosis and appropriate treatment i.e. only positive cases of uncomplicated malaria received ACTs

^b^ *OR* Odd Ratio

### Factors influencing type of antimalarial drugs prescribed

Majority of the providers in both public (66.7 %) and private (61.6 %) settings considered availability of the antimalarial medicines before prescribing a specific antimalarial medicine to the patients, *p* = 0.27 (Table 5). However, 64.8 % of health workers in public facilities and 36.5 % in private facilities considered the recommendations of the treatment guidelines before commencing treatment (*p* < 0.0001). Drug promotion was reported among 8.8 % health worker in public and 45.8 % in private health facilities (*p* = 0.000). Patient preferences, need to make profit, and patients request were mentioned by 18.5 %, 10.2 % and 5.6 % respectively in private health facilities, to influence treatment provided compared to 3.7 %, 0.5 %, and 0.9 % among those in public health facilities, respectively (*p* < 0.05).

**Table 5:** Factors influencing type of antimalarial drugs prescribed by health workers

	Public *N* = 216 n (%)	Private *N* = 216 n (%)	Total *N* = 432 n (%)	OR (95 % CI)	*P*-value
Drugs availability	144 (66.7)	133 (61.6)	277 (64.1)	1.25 (0.8–1.9)	0.27
Existing national malaria treatment guidelines	140 (64.8)	79 (36.5)	219 (50.7)	3.19 (2.2–4.7)	0.000
Drug promotion by manufacturers	19 (8.8)	99 (45.8)	118 (27.3)	0.11 (0.1–0.2)	0.000
Idea of what the consumer prefers	8 (3.7)	40 (18.5)	48 (11.1)	0.16 (0.8–0.4)	0.0002
Need to make profit	1 (0.5)	22 (10.2)	23 (5.3)	0.04 (0.0–0.3)	0.0001
Demand by patients	2 (0.9)	12 (5.6)	24 (5.6)	0.15 (0.0–0.7)	0.01

## Discussion

The study showed high level of awareness of national treatment guideline among health workers in public and private settings. Malaria RDT was more available in the public health facilities and also was its use for parasitological diagnosis of malaria. Presumptive diagnosis of malaria was higher among private healthcare workers and the main reason for non-usage of mRDT was perceived non reliability of mRDT results. The recommended ACTs were available in high proportion in both private and public health facilities but monotherapy antimalarial medicines, such as artesunate, amodiaquine and chloroquine were significantly more available in the private facilities. Adherence to national diagnosis and treatment guidelines was significantly higher among public healthcare workers (60.6 %) compared to those in private facilities (27.3 %). The type of antimalarial available in facility stocks influenced treatment prescription in both healthcare settings. Drug promotion by manufactures (45.8 %) had a major influence on private healthcare workers’ prescription practice.

This study findings indicate that high level of awareness of national treatment guideline was demonstrated among health workers in both public (98.1 %) and private (94.8 %) settings This is in contrast to a study done in Tanzania which revealed that 15.5 % of health care workers were aware of the country’s guidelines [17]. Twenty percent of the health workers in private sector had received training on case management of malaria within the last 3 years. This proportion is less than that reported in a study done in Kenya where 46 % of private health workers received training on malaria treatment [9]. The health worker’s knowledge of malaria case management was generally lower in the private sector than in the public sector. This is similar to findings from other studies which showed that health workers’ knowledge of drugs and dose regimens, particularly in the private sector, is often poor [18].

Rapid diagnostic test kits were more available in the public health facilities (82.0 %) than private health facilities (19.2 %). This finding underscores the need to scale-up mRDTs among private health facilities since a majority (about 60 %) of the populace in the State patronize them [19]. Currently, mRDTs are supplied free of charge by government to public primary health facilities and this may have accounted for the better availability of mRDT in public facilities. Majority of the health workers interviewed from public health facilities also used mRDTs (85.2 %) compared to health workers in private setting (32.9 %). Some of the limitations of the use of mRDTs as noted by the health workers in private setting include cost and availability of mRDT. This is likely to promote treatment based on presumptive diagnosis of malaria by health workers in private health facilities. A positive finding however, was that health workers in both public and private facilities knew that mRDT could be affected by temperature and humidity. This is important for maintaining the quality of mRDT in the facilities.

The availability of the recommended ACT in the public (94.2 %) and private (79.9 %) sectors is commendable and shows the level of confidence the health facilities has in ACT and to some extent, the effect of initiatives such as affordable medicine facility for malaria (AMFm) and private sector co-payment mechanism that subsidize cost of ACTs in private and support for ACTs in public sector from Global Fund grants, United States Presidents’ Malaria Initiatives (PMI) and United Kingdom Aid for International Development (UKAID). This is a positive findings in the push to improve the case management of malaria in the State. It also indicates the progress made over the years when compared to results of a study on malaria control practice done in 2010 which showed that less than a fifth of the primary and secondary health facilities used the recommended ACT [20]. Artemisinin-based combination therapy (ACTs) especially the recommended first-line groups in the national treatment guidelines AL and co-formulated artesunat amodiaquine (AA) were readily available in public facilities. This could be attributed to the fact that ACTs donated by grant/donors are currently supplied free of charge for treatment of all parasitological confirmed malaria cases in public primary health facilities in the State (Ogun State Malaria Activities Report, 2012). Overall the availability of ACTs were lower in private sector compare to public sector but were more available in both types of facilities than mRDTs and microscopy. However, the fact that ACTs were readily available and were used, suggest that some patients particularly at private health facilities may be treated with ACTs without laboratory diagnosis. Parasitological diagnosis is a component of malaria case management, the first step without which adherence to national guidelines is incomplete. Improper and abusive use of ACTs without confirmatory diagnosis will result in negative clinical and economic impact [21].

The findings of this study also revealed that monotherapies, either as oral artemisinin-based (Artesunate) or non-artemisinin-based (Amodiaquine) were in stock for use in a sizeable proportion (58.3 % and 39.4 %), respectively in the private facilities. The use of artemisinin-based monotherapy is contrary to national policy and portend potential risk of parasite developing resistance to the medicine as a result of its short half-life [2]. Non-artemisinin monotherapies, typically chloroquine (26.9 %) which has been proscribed was significantly more available for use in private health facilities compared to public health facilities and were usually sold at a much lower price than ACTs. Given the relative affordability and accessibility of non-artemisinin therapies, health workers at private health facilities are likely to choose incorrect and ineffective anti-malarial drugs for treatment of malaria. This finding is similar to results of a study done in Anambra, southeastern Nigeria which assessed the quality of antimalarial drugs provided by public and private health care providers and found that monotherapies such as chloroquine, SP, quinine, artesunate and dihydroartemisinin were still widely used for treatment of malaria [15].

Health workers who used any of the malaria laboratory diagnostic method (RDT or microscopy) were significantly more likely to adhere to national treatment guideline in both the public and private health facilities. This finding was similar to study by Marycelina et al. in Tanzania where health workers overall adherence to national treatment guideline was 90.5 % [22]. The study failed to find an association between case management training of public and private health workers and adherence to guidelines. This was consistent with other studies showing that being trained does not necessarily translate into correct behavior [23, 24]. Availability of antimalarial drugs was observed to be a major factor that affected treatment prescription of both public and private health setting. This implied that health workers are sometime constrained to prescribe the antimalarial drugs available in the facilities even if they are not the recommended ones. This supports the results of earlier studies that found that prescribing patterns are more likely to follow availability of antimalarial drugs [25]. In this study, drug promotion by manufacturers to the providers was a significant factor that influences drug prescribed by health workers in private setting. This is worrisome because it could lead to supplier-induced demand and prescription of unnecessary drugs thereby worsening the economic burden of the disease on the consumers and predisposing to development of drug resistance if the drugs are wrongly used. The factors found to influence health workers’ malaria treatment prescription behavior constitute focus to targets in the planning of intervention to improve the treatment of malaria in health facilities in the state.

### Study limitations

The interviews and discussions were conducted at the heath facilities and the questionnaire captured self-reported information and relied primarily on respondents providing the right information. There might have been some reporting bias by respondents due to fear of disclosing information that respondents believed could tarnish the image of their health facility. Hence there is probably the tendency to overestimate adherence in this study since this is a desirable outcome. However this was minimized by ensuring that participants were interviewed in a secluded place whereby a high degree of privacy was observed and reassured that the information obtained will be used solely for research purposes.. Some health facilities data records were incompletely filled, and this affected the completeness of the information obtained using the health facility inventory checklist.

## Conclusion

The findings of this study found close similarity in the level of awareness of the national malaria treatment guidelines, but a remarkable difference in compliance to appropriate case management of uncomplicated malaria between public and private health facilities in the State. In general, health workers in both public and private sectors had fairly good knowledge on the national malaria treatment guidelines for case management of uncomplicated malaria. Majority of health workers in private setting were not using RDTs because of perceived unreliability, high cost and irregular supply. Also presumptive diagnosis and use of antimalarial monotherapy drugs are still common practices among providers in private health setting. This may increase the risk of development of parasite resistance to the currently effective anti-malarial medicines and treatment failures, thereby undermining the goals of malaria treatment guidelines/policy. Availability of drugs influenced malaria treatment prescription behavior of providers in both health settings. However drug promotion by manufacturers may influence prescribing practices among private providers. Therefore, in order to improve the implementation process of the national malaria diagnosis and treatment guidelines, there is need to intensify strategies to enhance appropriate malaria case management in private sector and address factors influencing adherence to national guideline. Provision of mRDT and ACTs by the government to private health facilities as done for the public sector will help to ameliorate the influence of drug manufacturers.

## Abbreviations

AA: Artesunate amodiaquine, AMFm, Affordable medicines facility-malaria
ACT: Artemisinin based combination therapy
AL: Artemether lumenfantrine
CHEW: Community health extension workers
DSNO: Disease Surveillance and Notification Officer
FMOH: Federal ministry of health
IDSR: Integrated disease surveillance and response
LGA: Local Government Area
mRDT: Malaria rapid diagnostic test
NDHS: National demographic health survey
NMEP: National malaria elimination programme
PHC: Primary health care
RBM: Roll back malaria
SFH: Society for family health
SMOH: State Ministry of Health
SP: Sulphodoxine pyrimethamine
SuNMaP: Support for national malaria program
UKAID: United Kingdom Aid for International Development
WHO: World Health Organization
